# Antibacterial, Antifungal, and Wound-Healing Activities and Chemical Characterization of Propolis from *Apis mellifera* in Michoacan, Mexico

**DOI:** 10.3390/molecules30193880

**Published:** 2025-09-25

**Authors:** Ana Bertha Hernandez-Hernandez, Mario Rodriguez-Canales, Pilar Dominguez-Verano, Uriel Nava-Solis, Marco Aurelio Rodriguez-Monroy, María Margarita Canales-Martinez

**Affiliations:** 1 Laboratorio de Farmacognosia, UBIPRO Facultad de Estudios Superiores Iztacala UNAM, Tlalnepantla de Baz 54090, Edomex, Mexico; ana.b.hdez@iztacala.unam.mx (A.B.H.-H.); mario.rodcan09@gmail.com (M.R.-C.); biol.navasolis@gmail.com (U.N.-S.); 2 Laboratorio de Investigación Biomédica en Productos Naturales, Carrera de Medicina Facultad de Estudios Superiores Iztacala UNAM, Tlalnepantla de Baz 54090, Edomex, Mexico; pilardomver@hotmail.com (P.D.-V.); dr.marcorodriguezmonroy@gmail.com (M.A.R.-M.)

**Keywords:** propolis, bee, *Apis mellifera*, antibacterial, antifungal, wound healing

## Abstract

The aim of this study was to evaluate the antibacterial and antifungal activities, wound-healing efficacy, and chemical characteristics of hexanic, chloroformic, and methanolic extracts of propolis from Michoacan, Mexico. Antibacterial activity was determined using Gram-positive and Gram-negative bacteria, antifungal activity was determined using yeast and filamentous fungi and wound-healing efficacy was determined using the tensiometric and histological methods in mouse skin. Antioxidant capacity, phenols, and total flavonoids were quantified. Propolis was subjected to high-performance liquid chromatography (HPLC-DAD), high-performance liquid chromatography–mass spectrometry (HPLC-TOF-MS), and gas chromatography–mass spectrometry (GC-MS). The methanolic extract showed the best antibacterial activity, and the most sensitive bacteria was *Staphylococcus aureus*. For antifungal activity, yeasts and filamentous fungi showed sensitivity to the methanolic extract, with *Candida albicans* and *Trichophyton mentagrophytes* being the strains with the highest sensitivity to the extract. Regarding wound-healing efficacy, when using the tensiometric method, the methanolic extract presented the highest efficacy, surpassing the positive control (Recoveron). In the histological evaluation, the methanolic extract provided more resistance to the wound and demonstrated an antioxidant capacity of 12.23 µg/mL, a total phenolic content of 580 mg GAE/g, and a total flavonoid content of 12.35 mg QE/g. In the chemical analysis, flavanols, flavones, and flavanones were identified.

## 1. Introduction

Propolis is a material produced by bees (*Apis mellifera* L.); bees use their jaws to collect resinous particles from the buds, shoots, and petioles of the leaves of different plants, which are then mixed with wax and their salivary secretions to form propolis [1]. It is used for the construction of the hive, to maintain stable humidity and temperature throughout the year, and to seal cracks. It is also used to cover the corpses of enemies that have entered the hive (beetles, rodents, lizards, etc.), which are embalmed, thus preventing their decomposition [2]. At high temperatures, it is soft, flexible, and sticky, while after cooling, it becomes hard and brittle [3].

Propolis has been used in traditional medicine for many years due to the various biomedical properties attributed to it, such as antibacterial, antifungal, antiviral, anesthetic, antiulcer, immunostimulant, hypotensive, cytostatic, antioxidant, phytoinhibitory, anticariogenic, and regenerative or wound-healing properties [1].

The antibacterial and antifungal activities of propolis are very important due to the current problem of antimicrobial resistance and the search for new alternatives. When an infection by a pathogenic microorganism occurs, the body reacts to the pathogen through an inflammatory response, which generates oxidizing substances to inhibit its development, but this also affects the cells themselves; thus, it is noteworthy that propolis also has antioxidant properties, and all of these properties contribute to good wound healing [4].

These properties of propolis are due to its chemical composition, which consists of a mixture of polyphenols, including flavonoid aglycans, phenolic acids, phenolic aldehydes, and ketones. To date, more than 300 compounds have been identified in propolis, with phenolic acids, esters, aromatic aldehydes, coumarins, and flavonoids accounting for a high percentage [5]; however, research on propolis in different geographical areas has shown great differences in its chemical composition and biomedical activity due to the combination and synergy of the different compounds that it contains [6].

Mexico is a mega-diverse country and, due to its high biodiversity, the chemical composition of propolis differs depending on the geographical area and the floral diversity of the location at which it is collected; for this reason, its medicinal properties may also vary [1]. Beekeepers use propolis to make tinctures based on ethyl alcohol, but there are still no regulations or standards for the concentrations that should be used for this procedure. Thus, our research group has been working with several beekeepers in the country, analyzing a large number of propolis samples and observing differences in both the medicinal and chemical compositions. For this reason, the objectives of this work are, on the one hand, to give scientific support to a propolis sample from the state of Michoacán, evaluating several biomedical properties and its chemical composition, and, on the other hand, to reaffirm the importance of this bee product because, despite the fact that beekeeping has existed for a long time in our country, interest has largely focused on honey, resulting in the neglect of propolis, which is very important due to its medicinal properties.

## 2. Results

### 2.1. Organoleptic Characteristics

**Figure 1:**
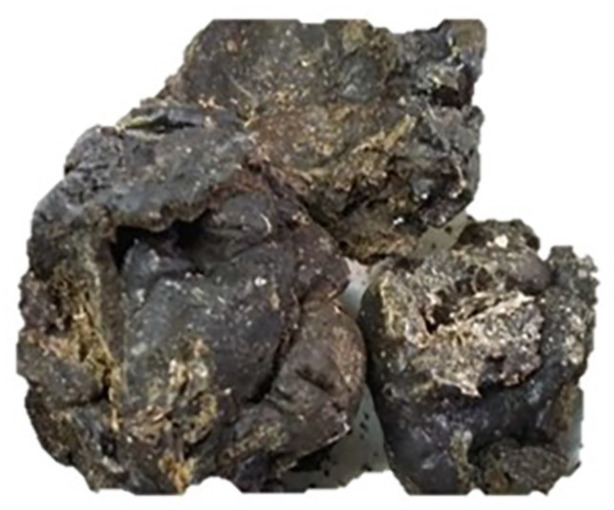
Raw propolis from Michoacan, Mexico.

The organoleptic characteristics of raw propolis obtained from Michoacan were determined; 10 people from our work team participated in the characterization, and they agreed with all the organoleptic characteristics (Table 1 and Figure 1).

### 2.2. Propolis Sample

A total of 105.50 g of raw propolis was collected, and three extracts were obtained, namely, methanolic, chloroformic, and hexanic extracts; additionally, the yield of each extract was calculated. The highest yield was obtained for the chloroformic extract (Table 2).

**Figure 2:**
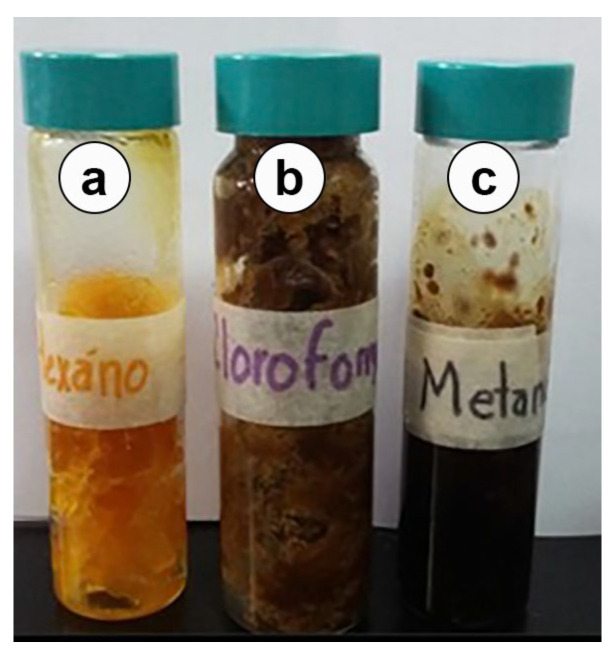
Extracts of propolis from Michoacan, Mexico. (**a**) Hexanic, (**b**) Chloroformic, (**c**) Methanolic.

The hexanic extract showed a yellow color and a consistency like wax, the chloroformic extract was dark brown and had a resinous smell, and the methanolic extract was black and had a resinous smell (Figure 2).

### 2.3. Antibacterial Activity

Table 3 shows the antibacterial activity results (inhibition halos, MIC, and MBC) of the propolis extracts. The methanolic extract showed inhibition halos with the greatest diameter against the Gram-positive and Gram-negative bacteria, followed by the hexanic and chloroformic extracts. The strains most sensitive to the extracts were *S. aureus*, *E. coli*, *E. aerogenes*, and *S. typhimurium.* There were significant differences in effectiveness between the extracts (*p* < 0.0001), between the bacterial strains (*p* < 0.0001), and between the extract–strain interactions (*p* < 0.0001). Each assay was performed in triplicate. The data show the media and standard deviation. The chloramphenicol concentration was 25 µg.

In the determination of the minimum inhibitory concentration (MIC) and the minimum bactericidal concentration (MBC), the methanolic extract presented the lowest values and the best antibacterial activity, and the most sensitive strains were *S. aureus* and *E. aerogenes*.

Based on the MIC and MBC results obtained, the strains most sensitive to each extract were selected to obtain bacterial growth curves.

**Figure 3:**
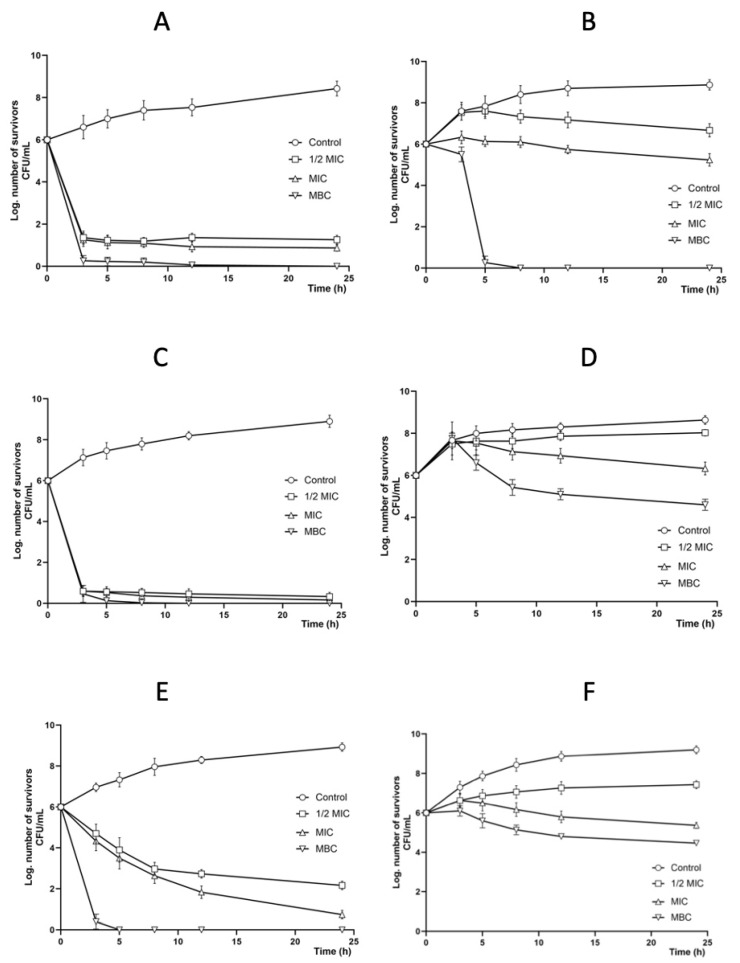
Effect of propolis extracts from Michoacan on the bacterial growth curve. (**A**): Methanolic extract on *S. aureus* CUSI-IZTA. Control: bacteria without any extract; 1/2 MIC: 1 mg/mL; MIC: 2 mg/mL; MBC: 4 mg/mL. (**B**): Methanolic extract on *E. aerogenes* FES-C. Control: bacteria without any extract; 1/2 MIC: 1 mg/mL; MIC: 2 mg/mL; MBC: 4 mg/mL. (**C**): Hexanic extract on *S. aureus*. Control: bacteria without any extract; 1/2 MIC: 1 mg/mL; MIC: 2 mg/mL; MBC: 4 mg/mL. (**D**): Hexanic extract on *S. typhimurium* CUSI-IZTA. Control: bacteria without any extract; 1/2 MIC: 5 mg/mL; MIC: 10 mg/mL; MBC: 20 mg/mL. The MBC only decreased bacterial growth. (**E**): Chloroformic extract on *S. aureus* CUSI-IZTA. Control: bacteria without any extract; 1/2 MIC: 5 mg/mL; MIC: 10 mg/mL; MBC: 20 mg/mL. (**F**): Chloroformic extract on *E. coli* CUSI-IZTA. Control: bacteria without any extract; 1/2 MIC: 5 mg/mL; MIC: 10 mg/mL; MBC: 20 mg/mL.

The methanolic extract had a bactericidal effect at the MBC, and, at the other concentrations (1/2 MIC and MIC), it caused a decrease in the populations of both bacterial strains *S. aureus* CUSI-IZTA (Figure 3A) and *E. aerogenes* FES-C (Figure 3B).

The hexanic extract had a bactericidal effect on *S. aureus* CUSI-IZTA at the three concentrations (1/2 MIC, MIC, and MBC) (Figure 3C). However, for *S. typhimurium* CUSI-IZTA, a decrease in the bacterial population was observed (Figure 3D).

Finally, the chloroformic extract had a bactericidal effect on *S. aureus* CUSI-IZTA at the MBC (Figure 3E), while for the strain *E. coli* CUSI-IZTA (Figure 3F), it only decreased bacterial growth at the three concentrations tested.

### 2.4. Antifungal Activity

As shown in Table 4, the *C. albicans* strains showed sensitivity to the methanolic extract, particularly the clinical case of *C. albicans*^1^, *C. tropicalis*^3^, and *C. glabrata*^1^. Nystatin served as the positive control (4 mg per disk). The strains *C. albicans* ATCC 10231 and *C. albicans*^1^ presented the lowest MFC values (5.0 and 2. 5 mg/mL). Hexanic and chloroform extracts showed no activity; the negative controls (disks impregnated with 10 μL of hexane, chloroform, and methanol) showed no inhibition halos.

For the growth curve, the two strains that presented the lowest MIC and MFC values were used: *C. albicans* ATCC 10231 and *C. albicans*^1^ (clinical case donated by FES-C)

**Figure 4:**
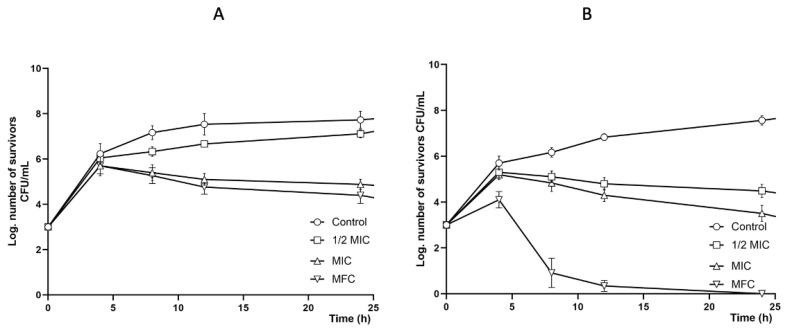
Activity of the propolis extracts on the yeast growth curve. (**A**): methanolic extract on *C. albicans* ATCC 10231. Control: yeast without any extract; MFC: 5.0 mg/mL, MIC: 2.5 mg/mL, and ½ MIC: 1.25 mg/mL. (**B**): methanolic extract on *Candida albicans*^1^ (clinical case donated by FES-C). Control: yeast without any extract; MFC: 2.5 mg/mL, MIC: 1.25 mg/mL, and ½ MIC: 0.625 mg/mL.

Figure 4 show the effect of the methanolic extract on the survival curves of *C. albicans* 10231 (4A) and *C. albicans*^1^ (4B). In both survival curves, all methanolic extract concentrations (MFC, MIC, and ½ MIC) reduced fungal growth; however, the growth of *C. albicans*^1^ was more affected than that of *C. albicans* 10231.

Table 5 shows the sensitivity of the filamentous fungi to the three propolis extracts. The methanolic extract presented the highest activity (+++). The strains *T. mentagrophytes* CDBB-H-1112 and *Fusarium moniliforme* were sensitive to the three extracts with a greater inhibition (+++). Ketoconazole served as the positive control (disk with 7 μg).

As the *T. mentagrophytes* strain was the most sensitive in the radial growth inhibition test, it was chosen to determine the FC_50_ and MFC in the three extracts. The MFC was 2.5 mg/mL for the methanolic extract, and it was 5 mg/mL for the chloroformic and hexanic extracts (Table 6).

### 2.5. Wound-Healing Activity

#### 2.5.1. Tensiometric Method

**Figure 5:**
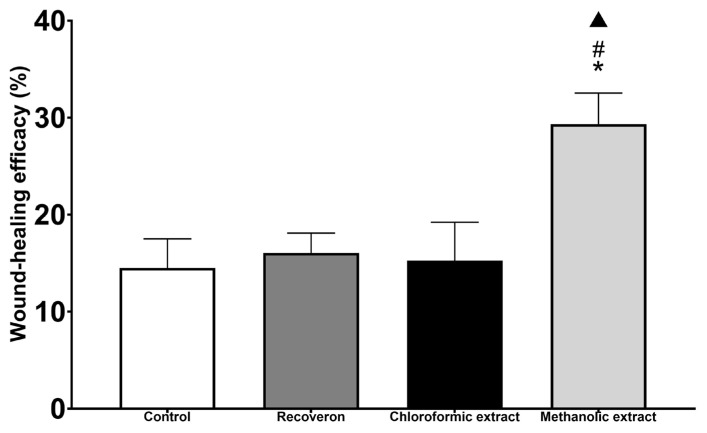
Wound-healing efficiency of propolis extracts determined using the tensiometric method. * Significant difference from the control group; # significant difference from the Recoveron group; ▲ significant difference from the chloroformic extract group.

Figure 5 shows that the methanolic extract group needed a greater force to open the wound (33%) than the Recoveron group (positive control) (16.07%) and the other two experimental groups: the control and chloroformic extract groups (14% and 15%, respectively). Thus, the methanolic extract contributes to more efficient wound closure. In addition, significant differences were observed between the methanolic extract group and the other experimental groups (*p* < 0.05).

#### 2.5.2. Histological Evaluation

The methanolic extract presented the greatest wound-healing force; thus, a histological evaluation of the injured area was carried out to observe the evolution of tissue repair.

To evaluate the wound-healing effect of the methanolic propolis extract, we analyzed the remaining wound area. The remaining wound area was analyzed in three experimental groups: control (no treatment), Recoveron, and methanolic extract treatment. The obtained values were expressed as a percentage of the wound area and compared using one-way ANOVA followed by Tukey’s multiple comparisons test.

**Figure 6:**
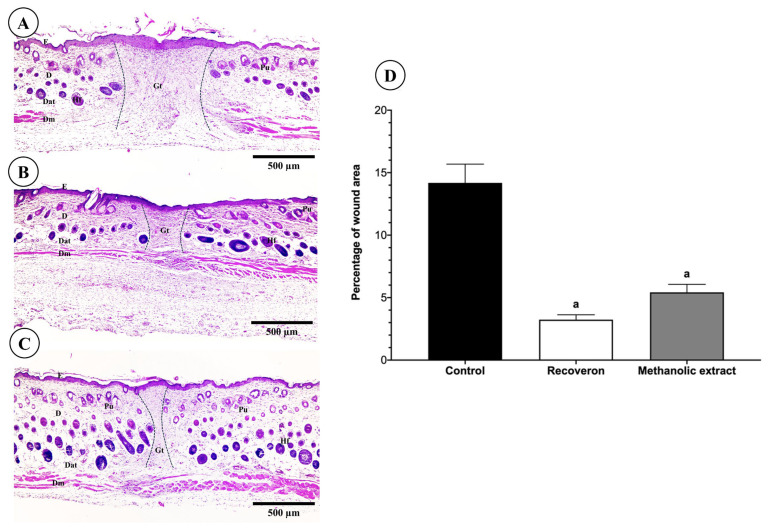
Effect of methanolic propolis extract on cutaneous wound healing. (**A**) Histological section of an untreated wound (control group) showing extensive disorganization of dermal tissue. (**B**) A section of a wound treated with Recoveron shows marked re-epithelialization and partial reorganization of skin appendages. (**C**) Section of a wound treated with methanolic propolis extract, displaying a continuous epidermis and reduced inflammatory infiltration. Images were stained with hematoxylin and eosin (H & E) and captured using a 4× objective. The remaining wound area was delimited with a dotted line. Epidermis (E), Dermis (D), Dermal adipose tissue (Dat), Dermal muscle (Dm), Granulation tissue (Gt), Hair follicles (Hf), and Pilosebaceous unit (Pu). (**D**) Shows the morphometric analysis of the remaining wound area. a: significant differences between the control and treated groups.

As shown in Figure 6, the control group (A) exhibited the largest remaining wound area (14.18 ± 0.79%), indicating a deficient healing process, which was also reflected histologically by a discontinuous epidermis and abundant inflammatory infiltrate. In contrast, the Recoverón treated group (B) showed a notable reduction in wound area (3.24 ± 0.79%; *p* * < 0.0001 vs. control), with a thinner but continuous epidermis and reorganization of skin appendages. Meanwhile, the methanolic propolis extract-treated group (C) also displayed a significant decrease in wound area (5.43 ± 0.79%; *p* < 0.0001 vs. control), along with re-epithelialized epidermis and partially restored dermal structure.

Statistical analysis revealed highly significant differences between the control group and both the Recoveron and methanolic propolis extract-treated groups (*p* < 0.0001 in both cases). However, no significant difference was observed between the Recoveron and methanolic extract groups, suggesting that the propolis extract exhibits an effect comparable to the reference treatment.

**Figure 7:**
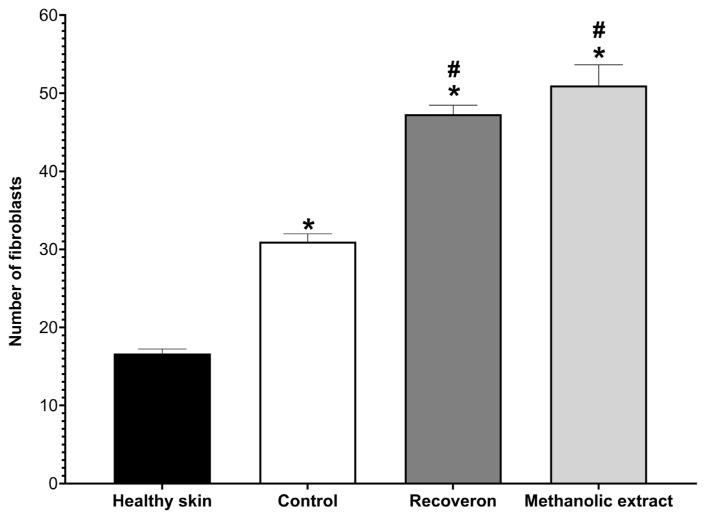
Average number of fibroblasts in each experimental group. * Significant difference from the healthy skin group; # significant difference from the control group (*p* < 0.05).

Finally, the number of active fibroblasts was counted (Figure 7). The healthy skin group presented the lowest value; this is because only when there is an injury do fibroblasts activate, differentiate from totipotent cells, and proliferate in the injured area. Therefore, the other experimental groups showed higher values, especially the positive control (Recoveron) and the methanolic extract of propolis, and they did not present significant differences between them; that is, they facilitated the rapid proliferation of fibroblasts, generating greater collagen synthesis.

### 2.6. Antioxidant Activity

The antioxidant capacity results are shown in Table 7. The methanolic extract presented a higher antioxidant capacity than the chloroformic and hexanic extracts. According to the antioxidant activity index (AAI) [7], the methanolic extract of propolis presented a value of 2.25; thus, its antioxidant activity is very strong. Quercetin was used as a positive control.

### 2.7. Total Phenol and Flavonoid Contents

Regarding the quantification of total phenols and flavonoids, the methanolic extract presented the highest concentration of these compounds (Table 8).

### 2.8. GC-MS, HPLC-DAD, and HPLC-MS Analyses

The hexanic extract was subjected to a GC-MS analysis, and three compounds were identified: eicosane, which was the most abundant compound, followed by heptacosane and hexacosane (Table 9) (File S1). The methanolic extract derivatized via GC-MS showed different carbohydrates (Table 10).

Through the HPLC-DAD analysis, three compounds were identified in the methanolic extract: pinocembrin, acacetin, and kaempferol. In the chloroformic extract, pinocembrin and acacetin were identified (Table 11) (File S1).

The three propolis extracts were subjected to an HPLC-TOF-MS analysis. Four compounds (chrysin, pinocembrin, naringenin, and acacetin) were identified in the methanolic and chloroformic extracts; in the hexanic extract, only three compounds were identified (chrysin, pinocembrin, and acacetin) (Table 12) (File S1).

## 3. Discussion

Propolis is defined as a natural resinous mixture, and it is produced by honey bees (*Apis mellifera*) using secretions collected from trees and herbaceous plants or resins, mucilage, and gums from the flowers, fruits, branches, stem, and leaves of different plants [8]. Propolis has been known and used as a medicinal product since ancient Greek, Roman, and Egyptian times. The complex composition of propolis allow this natural product to exert a broad spectrum of pharmacological activities [9].

The organoleptic characteristics of raw propolis from Michoacan (Table 1) are within the provisions of the Official Mexican Standard (2017) [10] and are essential for determining its quality and effectiveness as these characteristics are related to its chemical composition and medicinal benefits [10].

The chemical characterization of propolis from Michoacán was addressed using three complementary techniques: GC-MS, HPLC-DAD, and HPLC-MS. This approach allowed for the identification of volatile compounds and higher molecular weight flavonoids, strengthening the interpretation of the observed biological results (File S1).

The analysis by GC-MS revealed differences between the hexane and methanol fractions. Long-chain hydrocarbons such as eicosane (21.205%), heptacosane (10.692%), and hexacosane (5.802%) were identified in the hexane fraction (Table 9), which are non-polar compounds typically found in waxes, lipids, and plant extracts like propolis. Although these compounds are not typically considered bioactive, they have been reported in other propolis studies. They could contribute to the observed antimicrobial activity, mainly due to their ability to alter the permeability of bacterial membranes [11].

In contrast, the derivatized methanolic fraction showed the presence of carbohydrates such as arabinofuranose (18.2%), β-D-Lyxopyranose (0.474%), D-xylose (1.502%), inositol (0.867%), and 6-O-methyl-β-D-glucopyranose (9.531%) (Table 10). Most of these sugars do not exhibit documented direct biomedical activity; however, their presence may be related to structural roles, stabilization processes, or as byproducts of plant metabolism. In contrast, inositol has been associated with antioxidant effects, participating in the regulation of glucose metabolism and cell signaling, contributing to the reduction in oxidative stress and presenting regenerative effects on tissues, which could be related to the results obtained in wound healing [12].

Three flavonoids were identified in the methanolic and chloroform fractions using HPLC-DAD: pinocembrin, acacetin, and kaempferol (Table 11). The methanolic fraction presented all three compounds, while the chloroform fraction showed only pinocembrin and acacetin. These flavonoids have been extensively documented for their antimicrobial, antioxidant, and anti-inflammatory activity [1,3,4].

Kaempferol, present only in the methanolic fraction, has been shown to inhibit biofilm formation in Staphylococcus aureus by regulating genes such as clfA, clfB, fnbA, fnbB, and sarA [13]. This may explain this strain’s high sensitivity in antibacterial assays.

The differences observed in the retention times of pinocembrin and acacetin between the CE (chloroform) and ME (methanol) fractions, as shown in Table 11, highlight the influence of extract composition on chromatographic behavior, despite analyzing the same compounds. This variation is likely due to the distinct chemical profiles of each fraction, which modulate the interaction of flavonoids with the stationary phase. Parameters such as polarity, viscosity, local pH, and matrix interferences can significantly alter retention dynamics. These phenomena are well documented in complex plant matrices and may affect chromatographic selectivity, resolution, and reproducibility. Therefore, the observed shifts in retention times reinforce the need to consider extract-specific characteristics when interpreting flavonoid profiles [14].

The HPLC-MS analysis confirmed the presence of four flavonoids in the methanolic and chloroform fractions: chrysin, pinocembrin, naringenin, and acacetin (Table 12). Three of the hexane fraction (chrysin, pinocembrin, and acacetin) were detected, suggesting that even the least polar extracts can retain relevant bioactive compounds. Naringenin, present in the methanolic fraction, has been reported as a modulator of inflammation and oxidative stress, and its presence could be related to the observed efficacy in wound healing and in reducing the wound area [15].

The observed differences in detecting kaempferol, chrysin, and naringenin can be attributed to the inherent differences between HPLC-DAD and HPLC-MS techniques.

While HPLC-DAD relies on UV absorbance, which can limit the detection of compounds present in trace concentrations or generate spectral interferences in complex matrices, HPLC-MS offers greater sensitivity and specificity, allowing for the identification of flavonoids even at low levels through mass-to-charge ratio (*m*/*z*) analysis. Moreover, the ionization efficiency in mass spectrometry can vary depending on the compound’s chemical structure and the sample’s environment, which influences the analytical response. The matrix effect can also cause signal suppression or enhancement in MS, or interferences in DAD, affecting the visibility of certain compounds in each fraction [16].

This underscores the importance of integrating complementary analytical techniques to achieve a comprehensive chemical profile, especially in complex natural products like propolis.

The propolis from Michoacán showed antibacterial activity against Gram-positive and Gram-negative bacteria; the methanolic extract yielded the best results, both in the agar diffusion assay and in the values of MIC and MBC. The most sensitive strains were *S. aureus* and *E. aerogenes*. Although several authors consider that MIC values lower than 1 mg/mL indicate high potency in crude extracts [17], the results obtained for Michoacán propolis exceed this value. Nevertheless, the results remain relevant, as the antimicrobial, antioxidant, and wound-healing activities observed can be attributed to the chemical complexity of propolis, the botanical variability of the collection region, and the presence of synergistic compounds that distinguish it from other propolis from other regions, making it unique.

Furthermore, factors such as the type of solvent used, extraction conditions, and the intrinsic resistance of each evaluated bacterial strain can affect the MIC values. Therefore, although the results are not considered highly potent according to strict criteria, they provide valuable preliminary evidence of the bioactive potential of propolis from Michoacán [18].

The antibacterial activity of propolis is related to its direct action on the microorganism, and it stimulates the immune system by activating the body’s natural defenses [19,20]. Propolis intervenes in the permeability of the cell membrane of the microorganism, the alteration of the membrane potential, and the production of adenosine triphosphate (ATP), as well as the decrease in bacterial mobility, and it can also act on the synthesis of nucleic acids.

However, it has been observed that propolis has greater activity against Gram-positive bacteria than against Gram-negative bacteria; this is due to the structure of the outer membrane of Gram-negative bacteria and the production of hydrolytic enzymes that decompose the active compounds of propolis [21,22,23].

The flavonoids pinocembrin, acacetin and kaempferol found in the chemical composition of propolis from Michoacan (Table 11 and Table 12), and several studies have demonstrated their antibacterial activity against *S. mutans*, *S. sobrinus*, *S. aureus*, *E. faecalis*, *L. monocytogenes*, *P. aeruginosa, Escherichia coli*, and *K. pneumoniae*, observing changes in membrane composition and cell lysis [24,25,26,27,28,29,30].

A pharmaceutical study showed that kaempferol is a potential antimicrobial agent and inhibits various pathogenic microorganisms. It was shown that kaempferol acts by inhibiting the activity of pathogens (*Staphylococcus aureus*) and hinders the anchoring of surface proteins, thereby reducing the adhesion of fibrinogen, which promotes biofilm formation [13,31]. Another possible mechanism of pathogen growth inhibition is related to the inhibition of gene expression involved in biofilm formation. Kaempferol reduces the gene expression levels related to biofilm formation, such as clump factors A and B (clfA and clfB), fibronectin binding proteins A and B (fnbA and fnbB), and staphylococcal accessory regulator A (sarA), in *Staphylococcus aureus* [13].

However, the methanolic extract of propolis from Michoacan showed the best results in terms of antifungal activity against the yeast strains of *C. albicans*, *C. tropicalis*, and *C. glabrata*, as well as against the filamentous fungi *Fusarium moniliforme* CDBB-H-265, *Trichophyton mentagrophytes* CDBB-H-1112, *Aspergillus niger* CDBB-H-179, and *Rizoctonia lilacina* CDBB-H-306. *Trichophyton mentagrophytes* was the strain most sensitive to the methanolic extract.

Various studies have shown that propolis acts on the cell membrane of fungi and induces cell death [32]. It also inhibits the activity of extracellular phospholipase, which leads to an attenuation of the adhesion of fungal cells to the epithelium; may influence the formation and integrity of the fungal cell wall; and may inhibit the morphological transformation of *C. albicans* [33,34].

Various studies have investigated the mechanism of action of propolis from different geographical regions, using concentrations different from those employed in this research with propolis from Michoacán. For example, Freires and collaborators [35] reported that a sample of ethanolic extract of propolis from Brazil was able to damage biofilms of *Candida* sp. at concentrations lower than 0.9 μg/mL, an effect attributed to the high presence of flavonoids such as kaempferol and quercetin. Similarly, Fernández-Calderón et al. demonstrated that a 70% ethanolic extract of propolis from Spain inhibited the formation of fungal biofilms at subinhibitory concentrations (0.1% and 0.05%) [36]. For his part, Gucwa [32] evaluated 50 ethanolic extracts of propolis (EEP) collected from apiaries in Poland against 69 clinical isolates of *Candida albicans*, observing satisfactory antifungal activity in most cases, with minimum fungicidal concentrations (MFC) between 0.08% and 1.25% (*v*/*v*). The eradication of biofilms in polystyrene microtiter plates (MBEC_50_) required concentrations between 0.04% and >1.25% (*v*/*v*). Among the compounds identified in these extracts are kaempferol, pinocembrin, acacetin, and naringenin, also present in Michoacán propolis.

Also, high efficacy was observed in eradicating biofilms formed by *C. glabrata* and *C. krusei* on PVC surfaces and silicone catheters. At subinhibitory concentrations, the EEP inhibited the morphological transformation of *C. albicans* from yeast to mycelium in liquid medium and mycelial growth in solid medium. A synergistic effect was evident between the EEP and the antifungals fluconazole (FLU) and voriconazole (VOR), reducing the minimum inhibitory concentrations (MIC) of both drugs by 32 to 256 times in the presence of EEP at concentrations as low as 0.02%. These findings support that the fungal cell membrane may be the most likely target of action of the EEP [32].

Regarding filamentous fungi, various studies have observed the antifungal activity of propolis. For example, Batac et al. tested the antifungal activity of propolis from the Philippines and found that it inhibited *Trichophyton mentagrophytes* (MIC = 0.08 g/mL) and *Microsporum gypseum* (MIC = 0.28 g/mL) [37]. Koç and Silici [38] evaluated the antifungal activity of an 80% ethanolic extract of propolis collected in Turkey, and the fungal strains used were *Trichophyton rubrum* and *Trichophyton mentagrophytes*. Both strains were sensitive to propolis (*T. rubrum:* MIC_50_ = 0.2 mg/mL; *T. mentagrophytes:* MIC_50_ = 0.2 mg/mL), and this activity was attributed to the presence of phenolic compounds [39].

These results agree with those obtained with Michoacan propolis as both yeasts and filamentous fungi were sensitive, and, although the propolis concentrations were different, it is important to mention that the properties of each propolis sample depend on its composition, method of collection, climatic conditions, and surrounding flora [35].

As previously observed, propolis from different regions contains compounds also present in the propolis from Michoacan, suggesting a certain chemical similarity despite different geographical and botanical origins. This phytochemical coincidence allows for comparisons regarding antimicrobial activity, although it is important to recognize that the composition of propolis can vary significantly depending on local flora and environmental conditions.

Michoacan propolis has healing activity; in the evaluation of wound-healing efficacy (Figure 5), the methanolic extract obtained a higher percentage (30%) than Recoveron (16%), which served as the positive control. Recoveron is a medication used for wound healing and is a tissue regenerator that contains acexamic acid, which participates in the protein action of collagen, regulating the production of fibroblasts and the arrangement of collagen fibers [40].

In the histological analysis (Figure 6), the area of the wound was observed and measured, the methanolic extract showed a significant decrease in the wound area, with re-epithelialized epidermis and partially restores dermal structure without inflammatory infiltrate, also, there were no significant differences with the Recoveron group, suggesting that the propolis extract exhibits an effect comparable to the reference treatment.

The wound-healing process is divided into three phases, namely, the inflammatory, proliferative, and remodeling phases, each of which lasts for a certain amount of time and involves the participation of various tissues and cell types. In the proliferative phase, a characteristic cell group is fibroblasts. Fibroblasts play a fundamental role in the wound-healing process, where they participate in the synthesis of collagen and elastin fibers, whereas in the remodeling phase, they contribute to the phagocytoses of collagen and extracellular matrix components [41].

In the histological analysis, the methanolic extract of propolis did not present significant differences from the Recoveron group in terms of the number of fibroblasts in the wound area (Figure 7); this means that the methanolic extract of propolis promotes skin wound healing possibly through anti-inflammatory and re-epithelialization mechanisms and promoting the migration and proliferation of fibroblasts to the wound area.

Although no significant difference was observed in fibroblast proliferation between the methanolic extract and the positive control (Figure 7), a higher tensile strength was recorded in the group treated with the methanolic extract (Figure 5). This finding suggests improved organization and maturation of the repaired tissue. Such a difference may be associated with enhanced extracellular matrix quality or modulation of inflammatory processes, rather than with fibroblast quantity.

Propolis from Michoacan contains a mixture of compounds that act synergistically, as most of them are anti-inflammatory, such as pinocembrin—which inhibits the production of proinflammatory cytokines (such as TNF-α and IL-1β) and contains factors such as nitric oxide, which are related to chronic inflammation [42]—or acacetin, which inhibits various proinflammatory enzymes, such as cyclooxygenase-2 (COX-2) and lipoxygenase [43]. Furthermore, kaempferol, naringenin, and chrysin, which were found to be present in Michoacan propolis, have anti-inflammatory properties [44,45,46,47,48].

For this reason, it is likely that the inflammatory phase of the healing process is carried out in a more efficient way due to the presence of these secondary metabolites and leads to the proliferative phase, as flavonoids stabilize the lysosomal membrane, preventing the loss of fluids at the cellular level, reducing inflammation, allowing for the proliferation of fibroblasts in a short time, and contributing to more efficient closure of the wound [41,49,50].

Propolis has been used since ancient times for the treatment of skin diseases, and, to date, its pharmacological potential for healing and repairing various types of wounds has been widely cited in the literature [51].

Hozzein et al. worked with propolis collected from Saudi Arabia and investigated the effects of the topical application of propolis on the healing and closure of diabetic wounds in a streptozotocin (STZ)-induced type I diabetic mouse model. The topical application of propolis significantly promoted wound closure in diabetic murine models; furthermore, a reduction was observed in the levels of IL-1β, IL-6, TNF-α, and MMP9, approaching normal values. Notably, compared to untreated diabetic mice, treatment with propolis significantly stimulated collagen synthesis in the damaged tissues, mediated by the activation of the TGF-β1/Smad2,3 signaling pathway [52].

As is well known, when an infection by bacteria or fungus occurs, or in the wound-healing process, various cell types produce reactive oxygen species, which act against the invading microorganisms and regulate various cell signaling pathways. At normal physiological concentrations, ROS play an important role in the regulation of cellular functions; however, at high concentrations, these molecules or a deficit in the antioxidant defense system, a condition known as oxidative stress, can cause serious damage at the cellular level, which can negatively affect the wound-healing process, resulting in the inflammatory phase being chronically maintained [49,53].

For this reason, the antioxidant capacity was evaluated, and the methanolic extract presented the best antioxidant capacity (12.23 µg/mL) compared to the chloroformic extract (1210.35 µg/mL).

The antioxidant capacity is directly related to the content of phenolic compounds in propolis. In propolis from Michoacan, the total phenols and flavonoids were quantified, and it was found that the content of these compounds was higher in the methanolic extract (580 mg AGE/g of sample and 12.635 mg QE/g). It is important to mention that the levels of these secondary metabolites depend on the geographical area and the flora surrounding the apiaries.

NOM-003-SAG/GAN-2017 [10] suggests a minimum of 5% (weight/weight) for phenolic compounds and 0.5% (weight/weight) for flavonoids. The methanolic extract of propolis from Michoacan not only meets the minimum established values but also surpasses them, as it contains 58% of phenols AGE/g and 1.235% of flavonoids QE/g.

Flavonoids have excellent antioxidant activity, as they scavenge free radicals and ROS, chelate metals, and prevent the oxidation of low-density lipoproteins (LDLs); however, this activity depends on their structure, especially the location and number of hydroxyl groups and the nature of the substituents on the aromatic rings [54].

Additionally, they inhibit oxidases, namely, lipooxygenase, cyclooxygenase, myeloperoxidase, and xanthine oxidase, thus avoiding the formation of reactive oxygen species. They also inhibit enzymes indirectly involved in oxidative processes, such as phospholipase A2, while they stimulate others with recognized antioxidant properties, such as catalase and superoxide dismutase [55].

Pinocembrin is considered a common component in various propolis samples and has a wide array of biological effects, including antioxidant, anti-inflammatory, anticancer, antifibrotic, neuroprotective, cardiovascular, and antimicrobial activities [56]. Flavanones, including pinocembrin, are generally weaker free radical scavengers than flavonols such as quercetin, which has additional hydroxyl groups on the B ring and conjugation between the A ring and the carbonyl group at C4, characteristics that promote the stabilization of the radical and the donation of electrons, unlike pinocembrin, which with a less conjugated structure and fewer hydroxyl groups, shows a more limited antioxidant capacity [57]. However, pinocembrin has shown strong antioxidant properties in different models via the induction of endogenous antioxidant capacity [15].

Nrf2 is a transcription factor that regulates the cellular redox balance by regulating the expression of antioxidant enzymes, e.g., glutathione peroxidase 1 (GPX1), thioredoxin reductase 1, NADPH-quinone oxidoreductase 1, glutathione-S-transferase, superoxide dismutase (SOD), catalase (CAT), peroxiredoxin, and heme oxygenase-1 (HO-1) [58,59]. In wound-healing models, Nrf2 promotes cell proliferation, angiogenesis, and extracellular matrix remodeling, thus contributing to a more efficient regeneration of damaged tissue [60].

Additionally, the activation of Nrf2 by pinocembrin has been evidenced in several disease models. For example, it has demonstrated protective effects in stroke, Parkinson’s disease, and Alzheimer’s disease [15]. Furthermore, pinocembrin protects against heart failure-induced cardiac dysfunction and remodeling in rats [61], and the flavonoid has also shown beneficial effects in liver fibrosis, all through the same mechanism as the activation of the Nrf2 factor [62].

## 4. Materials and Methods

### 4.1. Organoleptic Characteristics

Ten people participated in the organoleptic characterization, and the specifications of the Official Mexican Propolis Standard 2017 were considered (Table 13) [10].

### 4.2. Propolis Sample

Propolis was collected on November 16, 2016, from an apiary located in Cerro La Mole in the municipality of Zacapu, Michoacan de Ocampo (19°48′50″ N 101°47′26″ O). The predominant vegetation in this region includes oak–pine forest, pine forest, oak forest, and induced grassland, species commonly associated with the production of resins used by bees to make propolis [63].

Raw propolis (105.50 g) were cleaned to remove visible impurities and manually crushed to enhance solvent penetration. No specific analyses were performed to detect chemical residues, such as pesticides, heavy metals, or microbiological contaminants.

Sequential extraction was performed using three solvents of increasing polarity: hexane, chloroform, and methanol. A 1:10 (*w*/*v*) ratio was applied for each solvent, and the mixture was stirred continuously for 72 h at room temperature. Then, it was filtered using Whatman No. 5 paper. The solvent was removed under reduced pressure using a rotary evaporator. The resulting dry extracts were stored in amber glass bottles at −20 °C until use.

### 4.3. Antibacterial Activity Assay

Antibacterial activity was measured using the Kirby–Baüer disk diffusion agar method [64], following the guidelines of NORMA OFICIAL MEXICANA NOM-003-SAG/GAN-2017 [10]. The following strains were used: *Staphylococcus epidermidis* ATCC 12228, *Enterococcus faecalis* CDBB-B-1533, a *Staphylococcus aureus* clinical case donated by Clinica Universitaria de Salud Integral Iztacala UNAM (CUSI-IZTA), *Actinomyces viscosus* WFCC 449, *Pseudomonas aeruginosa* CDBB-B999, *Pantoea agglomerans* CDBB-B959, *Enterobacter aerogenes* CDBB-B-958, an *Enterobacter aerogenes* clinical case donated by the Laboratory of Microbiology of FES-Cuautitlán UNAM (FES-C), an *Escherichia coli* clinical case donated by CUSI-IZTA, a *Shigella flexneri* clinical case donated by CUSI-IZTA, a *Proteus miriabilis* clinical case donated by CUSI-IZTA, a *Salmonella typhimurium* clinical case donated by CUSI-IZTA, and *Salmonella typhi* CDBB-B-1111.

The bacterial inoculum was incubated in 10 mL Müeller–Hinton broth (BD DIFCO, BD Biosciences, Franklin Lakes, NJ, USA) at 37 °C for 24 h. The cultures were adjusted to a turbidity comparable to that of McFarland standard no. 0.5 (1.5 × 10^8^ CFU/mL). The microbial suspensions were plated on Müeller–Hinton agar plates (BD Bioxon-Mexico, Mexico City, Mexico). Disks with a 5 mm diameter (Whatman no. 5) were impregnated with 2.0 mg of the propolis extracts (hexanic, chloroformic, and methanolic); disks with 25 µg of chloramphenicol (Sigma-Aldrich, Darmstadt, Germany) were used as a positive control, and disks impregnated with 10 µL of each solvent used in the preparation of the extracts (hexane, chloroform, and methanol) were used as a negative control. The tests were performed in triplicate.

The minimal inhibitory concentration (MIC) and minimum bactericidal concentration (MBC) were estimated using the broth microdilution method. The concentrations used were 1, 2, 4, 8, 10, 15, and 20 mg/mL. The tubes were inoculated with a 1 × 10^5^ CFU/mL microorganism suspension. Inoculated plates were incubated at 36 °C for 24 h. After incubation, the plates were exposed to 0.08% tetrazolium chloride at 36 °C for 30 min, and each experiment was repeated three times [64]. The bactericidal or bacteriostatic effect of the extracts was determined based on bacterial growth curves. An assay was performed using the appropriate concentrations of the extract (corresponding to ½ MIC, MIC, and MBC).

### 4.4. Antifungal Activity Assay

#### 4.4.1. Yeast

The antifungal activity on yeasts was examined using the agar diffusion method, according to CLSI. The following strains were used: *Candida albicans* ATCC 10231, *Candida albicans* ATCC 14065, *Candida albicans* ATCC 32354, *Candida albicans* CDBB-L-1003, a *Candida albicans*^1^ clinical case donated by FES-C, a *Candida albicans*^2^ clinical case donated by Hospital Los Angeles, *Candida glabrata* CDBB-L-1536, *Candida glabrata* CBS 138, a *Candida glabrata* clinical case donated by CUSI-IZTA, *Candida tropicalis* CDBB-L-1098, a *Candida tropicalis*^1^ clinical case donated by Hospital Los Angeles, a *Candida tropicalis*^2^ clinical case donated by FES-C, a *Candida tropicalis*^3^ clinical case donated by Hospital Los Angeles, and a *Cryptococcus neoformans* clinical case donated by Laboratory of Mycology and Parasitology of School of Medicine UNAM.

Yeasts were cultured in 10 mL of liquid RPMI-1640 medium (Sigma-Aldrich, St Louis, MO, USA) at 36 °C for 48 h. The cell density was adjusted to 1 × 10^6^ CFU/mL using a Neubauer chamber. Aliquots of the suspension were plated on Mueller–Hinton agar supplemented with 2% glucose and 0.5 μg/mL of methylene blue. Sterile 5 mm disks (Whatman No. 5) were impregnated with 10 μL of the extracts (4 mg/disk). Disks with hexane, chloroform, or methanol were used as negative controls, while disks with 25 μg of nystatin (Sigma-Aldrich, St Louis, MO, USA) were used as a positive control. The plates were incubated at 36 °C for 24 h, and the inhibition zones (mm) were measured. All assays were performed in triplicate [65].

The determination of the minimum inhibitory concentration (MIC_50_) and the minimum fungicidal concentration (MFC) was performed using the broth microdilution technique [66]. The yeasts were cultured under the same conditions and adjusted to 1 × 10^3^ CFU/mL. Serial dilutions of the extract (0.6–25 mg/mL) were prepared in RPMI-1640 medium. Each microtube was inoculated with 50 μL of the yeast suspension and incubated at 36 °C for 48 h. After incubation, fungal growth was visually assessed, and aliquots were plated on Mueller–Hinton agar (2% glucose, 0.5 μg/mL methylene blue) to quantify CFUs. Microtubes with solvents were used as negative controls. The experiments were performed in triplicate [66,67].

To evaluate the kinetics of fungal growth, concentrations of the extract corresponding to ½ MIC, MIC, and MFC were used. Tubes were inoculated with 10 mL of RPMI-1640 medium and 100 μL of yeast suspension at a concentration of 1.5 × 10^5^ CFU/mL and incubated at 36 °C. At times 0, 4, 8, 12, 24, 28, 32, 36, and 48 h, samples of 50 μL were taken, diluted (1:100 and 1:10,000) with 0.85% NaCl isotonic solution, and plated on Petri dishes divided into three sections containing Mueller–Hinton agar (2% glucose, 0.5 μg/mL methylene blue). Each assay was repeated three times [68].

#### 4.4.2. Filamentous Fungi

The radial growth inhibition method was used. The strains *Fusarium moniliforme* CDBB-H-265, *Trichophyton mentagrophytes* CDBB-H-1112, *Aspergillus niger* CDBB-H-179, and *Rhizoctonia lilacina* CDBB-H-306 were used.

Petri dishes with 30 mL of potato dextrose agar (Bioxon, Mexico City, Estado de Mexico, Mexico) were inoculated, with a 5 mm diameter inoculum of each strain being placed in the center of each Petri dish.

After mycelial growth, sterile paper filter disks were placed at 0.5 cm from the colony’s edge. Each disk was impregnated with a 4 mg concentration of each extract. Ketoconazole (Sigma-Aldrich, St Louis, MO, USA) was used as a positive control (disk with 7 μg). The Petri dishes were incubated at 23 °C for 72 h until the colony growth enveloped the control group disks and crescents of inhibition formed around the disks containing the extracts [69]. Bioassays were performed in triplicate.

The radial growth inhibition method was used to determine the medium fungicidal concentration (FC_50_). The following concentrations were used for each extract: 1.25, 2.5, 5.0, 10.0, 15.0, and 20.0 mg/mL. Each of these concentrations were added to potato dextrose agar and placed in a 24-well culture dish, with three replicates performed for each concentration. In each well, a 0.5 mm diameter inoculum of each fungal strain was placed and incubated at 23 °C for 24 h. Subsequently, the colony area was measured, and colony growth inhibition was determined [69].

### 4.5. Laboratory Animals

For the evaluation of the healing activity, male CD1 strain mice (*Mus musculus*) aged seven weeks were used, which were handled in accordance with the official Mexican standard Nom-062-Zoo-1999 [70], which discusses the technical specifications for the production, care, and use of laboratory animals, approved by the Institutional Ethics Committee (CE/FESI/052019/1295).

### 4.6. Wound-Healing Activity

#### 4.6.1. Tensiometric Method

Seven-week-old male mice (*Mus musculus*) of the CD1 strain were handled following the Mexican federal regulations for animal use and care [70].

Wound-healing activity was examined using the tensiometric method based on measuring wound resistance to tension [71,72]. The methanolic and chloroformic extracts were used; they were dissolved with surgical gel (Ultra Sonic, Farmaceuticos Altamirano, S.A. de C.V., Mexico City, Mexico) to obtain a concentration of 10%, and 15 µL was administered throughout the wound. The experimental groups were (a) skin without a wound, (b) a wound without treatment, (c) Recoveron (Armstrong Laboratorios, Mexico City, Mexico) (positive control), (d) a 10%methanolic extract of propolis, and (e) a 10% chloroformic extract of propolis (*n* = 5 for each experimental group).

The mice were depilated using depilatory cream 24 h before the experiment, and then they were anesthetized via the inhalation of 5% isoflurane (Baxter, Deerfield, IL, USA). Afterward, a longitudinal cut of 1 cm was made in the depilated skin, considering the 3 skin layers (epidermis, dermis, and hypodermis). Treatments were applied topically every 12 h for 10 days. Later, the mice were euthanized using a CO_2_ chamber, and the tensile strength was taken as the load in grams necessary to reopen the wound. The results are expressed as a percentage of the tensile strength.

#### 4.6.2. Histological Evaluation

The histological technique was performed only with the methanolic extract. Male mice (*Mus musculus*) of the CD1 strain, with the same characteristics as those subjected to the tensiometric method, were used, and the same methodology was applied. The mice were euthanized using a CO_2_ chamber, and skin samples were obtained from the wound area for a histological evaluation. The tissue was processed routinely: 5 µm sections were cut with a microtome (ECOSHEL-315, Ecoshelworld, Pharr, TX, USA) and stained with hematoxylin and eosin. The experimental groups were skin without a wound, a control, and the methanolic extract.

For the observation of the histological sections, photographs of the injured area were taken with a Motic BA310E microscope at 10× and 40× magnifications. For the analysis, the area of the wound zone was measured using the ImageJ program (v.2.16.0/1.54p), and the fibroblast count were measured, all of which were identified based on their morphology and the presence of the central nucleus. The Motic Images Plus 2.0 program was used.

### 4.7. Antioxidant Activity (DPPH Free Radical Scavenging)

The antioxidant activity was determined via the decoloration of a methanol solution of DPPH (free radical, 2,2-diphenyl-1-picrylhydrazyl) [73]. Ninety-six-well ELISA plates were filled with the propolis extracts with concentrations ranging from 1 to 100 μg/mL and 100 μM of the DPPH solution. Quercetin (Sigma-Aldrich, Darmstadt, Germany) was used as a positive control, and all concentrations were examined in triplicate. The plates were incubated for 30 min at 37 °C, and the absorbance values were determined at 540 nm using an ELISA plate reader (Multiskan-Ex Thermo Scientific, Waltham, MA, USA). The antioxidant activity values were determined according to the following equation: % inhibition = [(absorbance of control-absorbance of sample)/absorbance of control] × 100. The concentration leading to 50% inhibition (SC_50_) was determined graphically.

### 4.8. Total Phenolic Content

The total phenolic content was determined via spectrophotometry (760 nm) based on a colorimetric oxidation–reduction reaction, using the Folin–Ciocalteu reagent (Hycel, Zapopan, Mexico) [74]. The mean of three readings was used to interpolate the gallic acid (Reasol, Mexico City, Mexico) curve (6.25, 12.5, 25, 50, 100, and 200 μg/mL), and the total phenolic content is expressed as mg of gallic acid equivalent (GAE)/g of extract.

### 4.9. Total Flavonoid Content

The Dowd method was used to determine the flavonoid content of the extracts [75]. The number of flavonoids was measured spectrophotometrically at 415 nm. A quercetin (Aldrich, Darmstadt, Germany) (1–100 μg/mL) calibration curve was used as the standard; the results are expressed as quercetin equivalents (QE)/g of extract.

### 4.10. Analysis of the Chemical Composition of the Extracts Using GC-MS, HPLC-DAD, and HPLC-TOF-MS

The hexanic extract was injected into GC-MS. The gas chromatograph model 6850 and the mass spectrometer model 5975 C (Agilent Technologies, Santa Clara, CA, USA) were used. An Agilent 19091S-433E column (30 m × 0.25 mm, 0.25 µm) was used. The initial temperature of the oven was 70 °C, and the heating ramp was 15 °C per minute until the maximum temperature of 290 °C was reached, which was maintained for 6 min. The mobile phase was helium. The injector temperature was 250 °C in split injection mode; the stream flow was 35cm/sec. The detector range of the mass spectrometer was 35–600 *m*/*z*, and 1 µL of sample was injected. The compounds were identified through a comparison with the NIST version 8.0 library database (National Institute of Standards and Technology, Gaithersburg, MD, USA).

Methanolic propolis extract (5 mg) was used to analyze the derivatized sample. Derivatization was carried out as follows: 50 μL of pyridine and 75 μL of bis(trimethylsilyl)trifluoroacetamide (BSTFA) with 1% trimethylchlorosilane (TMCS) were used, incubating the mixture for 1 h at 100 °C. Subsequently, the derivatizing agent was evaporated, and the residue was reconstituted in 500 μL of HPLC-grade hexane. 1 μL was injected in split mode. The analysis was performed on a 6850 gas chromatograph coupled to a 5975 C mass spectrometer (Agilent Technologies, Santa Clara, CA, USA), using an Agilent 19091S-433E column (30 m × 0.25 mm, 0.25 μm). The initial temperature was 100 °C, followed by an increase of 5 °C/min until reaching 300 °C. Helium was used as the carrier gas. The total analysis time was 40 min. Detection was performed in the 35 to 600 m/z range, with electron impact ionization at 70 eV and source temperature of 230 °C. Compound identification was compared with the NIST database version 8.0 (National Institute of Standards and Technology, Gaithersburg, MD, USA).

The methanolic and chloroformic extracts were injected at a concentration of 3 mg/mL with methanol-grade HPLC and a flow of 1 mL/min in the HPLC system model 1100 (Hewlett-Packard, Wilmington, DE, USA) with the diode array detector (DAD) 1100 ChemStation A0903. A Discovery C-18 (250 mm × 4.6 mm) column was used with a pressure of 269 bar and a temperature range of 22–23 °C. The separation was isocratic, using a mobile phase of water–acetonitrile–methanol (50:25:25) and phosphoric acid (0.1%), and the detector was used at a wavelength of 260 nm with a full scan of 200–400 nm. The components of each extract were identified according to a comparison of the retention time of each peak and the UV spectrum with those of the standards.

The following HPLC database standards were used: acacetin, baicalein, caffeine, catechin, catechol, chrysin, genistein, kaempferol, luteolin, myricetin, naringenin, naringin, genistein, pinocembrin, and quercetin. All standards were purchased from Sigma-Aldrich (St. Louis, MO, USA).

An HPLC-TOF-MS analysis of the hexanic, chloroformic, and methanolic extracts was performed using Agilent 1200 Infinity equipment, coupled to an Agilent 6230 TOF mass spectrometer with an Agilent Dual ESI source (ESISG14289023) (Agilent Technologies, Santa Clara, CA, USA) and Mass Hunter Workstation software, Version B.05.01, Build 5.01.5125, operating in negative ionization mode. The capillary voltage was 4000 V; the dry gas temperature was 250 °C, with nitrogen used as the dry gas at a flow rate of 6 L/min; the nebulizer pressure was 60 psi, and the fragment was 200 V; the MS range was 50–1300 *m*/*z*; and the MS acquisition rate was 1 spectra/s. A Kinetex 2.6µ, C1800Å (150 × 2.1 mm) column (Phenomenex) maintained at 25 °C and a two-line gradient mobile phase (solvents A and B) were used, where A = HPLC-grade water–HPLC-grade acetonitrile–formic acid (89:10:1) and B = HPLC-grade methanol–acetonitrile–formic acid (89:10:1). The first 3 min consisted of isocratic elution composed of 100% solvent A, followed by 3–11 min: 65% A, 35% B; 11–20 min: 55% A, 45% B; 20–35 min: 100% B. The flow rate was 0.2 mL/min, and the injection volume was 20 µL (3 mg/mL).

### 4.11. Statistical Analysis

The results are expressed as the mean ± standard deviation (SD). The data were analyzed using a one-way analysis of variance (ANOVA), with the Tukey–Kramer multiple comparison post hoc test (*p* < 0.05), using GraphPad Prism 6 software.

## 5. Conclusions

Due to the compounds identified in propolis from Michoacan and their reported biomedical activities, it is likely that propolis acts in an integral way in the wound-healing process. Firstly, it prevents any bacterial or fungal infection, decreases or neutralizes the free radicals generated in the wound area, prevents chronic inflammation from being triggered, and helps the proliferation of fibroblasts and collagen synthesis to start sooner, providing a greater closure force in a shorter period of time.

It is also important to mention that both the origin of propolis and the influence of geographical and environmental factors intervene in its chemical composition and pharmacological activities.

## Figures and Tables

**Table 1:** Organoleptic characteristics of propolis from Michoacan.

Organoleptic Characteristics	
Color	Brown
Smell	Resinous
Taste	Smooth balsamic
Consistency	Malleable

**Table 2:** Yield of the extracts obtained from the propolis from Michoacan.

Extract	Yield (g)	Yield (%)
Hexanic	19.20	19.55
Chloroformic	58.90	62.14
Methanolic	15.00	15.27

**Table 3:** Antibacterial activity of the three extracts of propolis from Michoacan.

Bacteria	Positive Control IH (mm)	Extract								
		Hexanic			Chloroformic			Methanolic		
		IH (mm)	MIC mg/mL	MBC mg/mL	IH (mm)	MIC mg/mL	MBC mg/mL	IH (mm)	MIC mg/mL	MBC mg/mL
1	12.0	6.6 ± 0.5	14.0	>20.0	6.0 ± 0.6	10.0	20.0	18.4 ± 0.2	2.0	4.0
2	17.0	7.2 ± 0.0	20.0	>20.0	7.0 ± 0.0	14.0	>20.0	8.4 ± 0.5	2.0	4.0
3	20.0	7.2 ± 0.0	2.0	4.0	6.4 ± 0.6	12.0	20.0	13.2 ± 1.1	2.0	4.0
4	22.5	6.4 ± 0.4	14.0	>20.0	7.2 ± 0.0	10.0	>20.0	16.8 ± 2.7	4.0	8.0
5	17.2	6.7 ± 0.4	16.0	>20.0	6.2 ± 0.4	8.0	20.0	18.4 ± 0.5	4.0	8.0
6	19.5	6.8 ± 0.4	20.0	>20.0	7.0 ± 0.4	10.0	>20.0	8.8 ± 0.8	2.0	4.0
7	23.2	6.7 ± 0.4	20.0	>20.0	7.0 ± 0.0	20.0	>20.0	16.0 ± 0.8	4.0	8.0
8	15.2	7.2 ± 0.0	20.0	>20.0	6.0 ± 0.2	15.0	20.0	17.4 ± 1.1	2.0	4.0
9	23.5	7.0 ± 0.0	20.0	>20.0	6.5 ± 0.5	10.0	>20.0	11.6 ± 0.5	8.0	10.0
10	22.4	7.4 ± 0.2	20.0	>20.0	7.0 ± 0.0	20.0	>20.0	16.8 ± 1.7	8.0	10.0
11	17.5	8.3 ± 0.5	20.0	>20.0	6.2 ± 0.4	15.0	>20.0	16.4 ± 1.5	8.0	10.0
12	22.0	7.2 ± 0.0	12.0	>20.0	6.4 ± 0.2	20.0	>20.0	14.4 ± 1.0	8.0	10.0
13	20.6	7.2 ± 0.0	20.0	>20.0	7.0 ± 0.0	10.0	20.0	16.4 ± 0.8	4.0	8.0

1. *Staphylococcus epidermidis* ATCC 12228, 2. *Enterococcus faecalis* CDBB-B-1533, 3. *Staphylococcus aureus* clinical case donated by Clinica Universitaria de Salud Integral Iztacala UNAM (CUSI-IZTA)*,* 4. *Actinomyces viscosus* WFCC 449, 5. *Pseudomonas aeruginosa* CDBB-B999, 6. *Pantoea agglomerans* CDBB-B959, 7. *Enterobacter aerogenes* CDBB-B-958, 8. *Enterobacter aerogenes* clinical case donated by Laboratory of Microbiology of FES-Cuautitlan UNAM (FES-C), 9. *Escherichia coli* clinical case donated by CUSI-IZTA, 10. *Shigella flexneri* clinical case donated by CUSI-IZTA, 11. *Proteus miriabilis* clinical case donated by CUSI-IZTA, 12. *Salmonella typhimurium* clinical case donated by CUSI-IZTA, 13. *Salmonella typhi* CDBB-B-1111. The values of inhibition halos are expressed as the mean ± standard deviation (SD). The other parameters are reported as individual values.

**Table 4:** Antifungal activity of propolis on yeasts.

	Inhibition Halos (mm)			
Yeast	Nystatin	Methanolic Extract	MIC mg/mL	MFC mg/mL
*C. albicans* ATCC 10231	7.6 ± 1.1	6.3± 0.5	2.5	5.0
*C. albicans* ATCC 14065	11.3 ± 2.3	6.0 ± 0.0	1.2	10.0
*C. albicans* ATCC 32354	12.6 ± 1.5	6.0 ± 0.0	2.5	15.0
*C. albicans* CDBB-L-1003	11.0 ± 1.7	6.0 ± 0.0	15.0	25.0
*C. albicans* ^1^	10.0 ± 0.0	6.6 ± 0.5	1.2	2.5
*C. albicans* ^2^	12.3 ± 5.6	6.0 ± 0.0	15.0	25.0
*C. glabrata* CDBB-L-1536	7.6 ± 1.1	6.3 ± 0.5	1.2	25.0
*C. glabrata* CBS 138	10.0 ± 1.0	6.0 ± 0.0	15.0	25.0
*C. glabrata* ^1^	10.3 ± 3.0	6.6 ± 0.5	2.5	25.0
*C. tropicalis* CDBB-L-1098	10.3 ± 1.5	6.0 ± 0.0	2.5	25.0
*C. tropicalis* ^1^	11.0 ± 1.0	6.3 ± 0.5	1.2	15.0
*C. tropicalis* ^2^	9.6 ± 1.5	6.0 ± 0.0	1.2	15.0
*C. tropicalis* ^3^	11.0 ± 1.7	7.0 ± 1.7	2.5	15.0
*C. neoformans*	8.6 ± 2.3	6.0 ± 1.0	15.0	25.0

*Candida albicans*^1^: clinical case donated by FES-C; *Candida albicans*^2^: clinical case donated by Hospital Los Angeles; *Candida glabrata*^1^: clinical case donated by CUSI-IZTA; *Candida tropicalis*^1^: clinical case donated by Hospital Los Angeles; a *Candida tropicalis*^2^: clinical case donated by FES-C; *Candida tropicalis*^3^: clinical case donated by Hospital Los Angeles. Each assay was performed in triplicate. The results are expressed as the mean ± standard deviation (SD).

**Table 5:** Sensitivity test of the filamentous fungal strains to the three propolis extracts.

Strain	Ketoconazole	Methanolic	Chloroformic	Hexanic
*F. moniliforme* CDBB-H-265	+++	++	++	++
*T. mentagrophytes* CDBB-H-1112	+++	+++	++	++
*A. niger* CDBB-H-179	++	++	+	NA
*R. lilacina CDBB-H-306*	++	++	NA	++

NA indicates that there was no activity and that the mycelium grew on the sensidisc, + indicates low growth inhibition, ++ indicates moderate growth inhibition, and, +++ indicates total growth inhibition.

**Table 6:** Mean fungicidal concentration (FC_50_) and minimum fungicidal concentration (MFC) of propolis extracts on the *T. mentagrophytes* CDBB-H-1112 strain.

	Methanolic	Chloroformic	Hexanic
FC_50_ (mg/mL)	1.25	2.50	2.50
MFC (mg/mL)	2.50	5.00	5.00

**Table 7:** The antioxidant capacity of the propolis extracts.

Extract/Standard	IC_50_ μg/mL	AAI
Methanolic	12.23	2.25
Chloroformic	1210.35	0.02
Hexanic	1354.50	0.02
Quercetin	5.30	4.58
	Antioxidant Activity Index (AAI)	
Poor	AAI <0.50	
Moderate	AAI 0.50–1.00	
Strong	AAI 1.00–2.00	
Very strong	AAI >2.00	

**Table 8:** Total phenol and flavonoid contents of propolis extracts.

Biomolecule	Methanolic Extract	Chloroformic Extract
Phenols (mg AGE/g)	580.00	20.00
Flavonoids (mg QE/g)	12.35	3.65

**Table 9:** Compounds identified via GC-MS in the hexanic extract of propolis from Michoacan.

Compound	Retention Time (min)	Similarity (%)	Abundance in Hexanic Extract (%)
Eicosane	16.977	93	21.205
Heptacosane	23.445	91	10.692
Hexacosane	14.216	95	5.802

**Table 10:** Compounds present in the derivatized sample of Michoacan propolis methanolic extract identified via GC-MS.

Compound	Retention Time (min)	Abundance (%)
Arabinofuranose	11.083	18.196
beta-D-Lyxopyranose	11.950	0.474
D-Xylose	13.332	1.502
6-O-methyl-beta-D-Glucopyranose	13.456	9.531
Inositol	14.507	0.867

**Table 11:** Secondary metabolites identified via HPLC-DAD.

Compound	Retention Time (min)		λ Max (nm)	
	CE	ME	CE	ME
Pinocembrin	8.273	8.636	210, 228, 290, 330.	210, 230, 290, 332.
Acacetin	46.739	48.309	269, 336.	210, 268, 324.
Kaempferol	ND	6.984	-	266, 294, 366.

CE = chloroformic extract, ME = methanolic extract. ND = Not Detected.

**Table 12:** Secondary metabolites identified via HPLC-MS.

Compound	Retention Time (min)			Parent Ion (*m*/*z*)		
	HE	CE	ME	HE	CE	ME
Chrysin	29.649	29.732	29.817	253.0595	253.0611	253.0621
Pinocembrin	28.886	28.969	29.07	255.0753	255.0769	255.0781
Naringenin	21.884	22.018	22.086	271.0692	271.0721	271.0729
Acacetin	ND	31.458	31.542	-	283.0717	283.0725

**Table 13:** Parameters used to determine organoleptic characteristics of raw propolis.

Parameters	Characteristics
Color	Red, yellow-reddish, yellow-dark, chestnut green, brown, or black, varying according to its botanical origin.
Smell	Resinous (woody smell) or balsamic (smell of wax), depending on its botanical origin.
Taste	Variable, smooth balsamic, strong, or spicy, depending on its botanical origin.
Consistency	Malleable or rigid at room temperature, depending on its botanical origin.

## Data Availability

The original contributions presented in this study are included in the article/Supplementary Material. Further inquiries can be directed to the corresponding author.

## Supplementary Materials

The following supporting information can be downloaded at: https://www.mdpi.com/article/10.3390/molecules30193880/s1, File S1: Chromatograms.
