# Supplementary material for: Antibacterial, Antifungal, and Wound-Healing Activities and Chemical Characterization of Propolis from Apis mellifera in Michoacan, Mexico
Source: Molecules. 2025 Sep 25;30(19):3880. doi: 10.3390/molecules30193880 (PMC12526293; doi:10.3390/molecules30193880)
Supplement: Supplementary file 1 [file molecules-30-03880-s001.zip › molecules-3820836-supplementary.pdf]

GC-MS

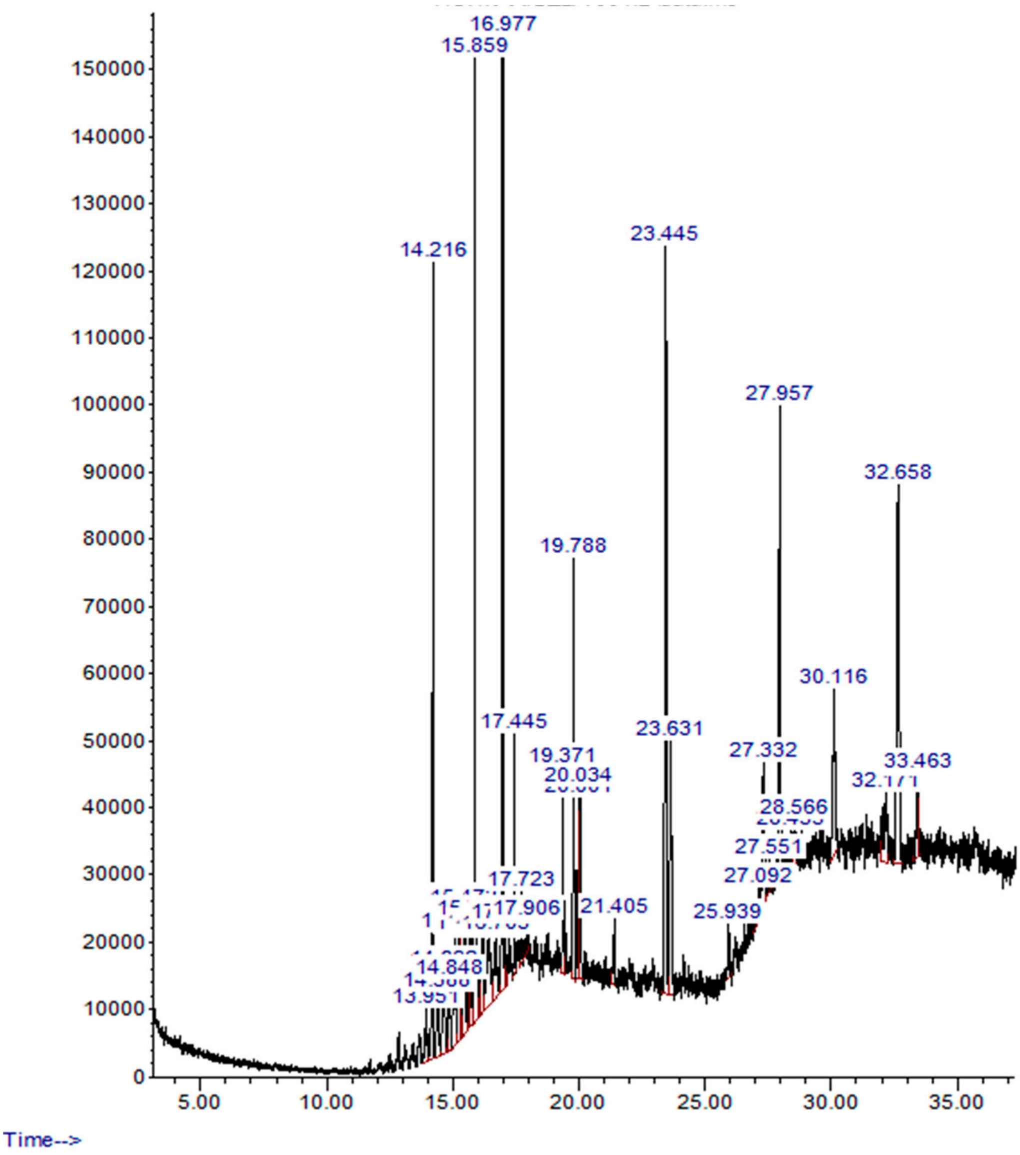

Chromatogram obtained by gas chromatography coupled with mass spectrometry (GC-MS) of the hexanic extract of propolis from Michoacan. The gas chromatograph model 6850 and the mass spectrometer model 5975 C (Agilent Technologies Santa Clara, CA, USA) were used. An Agilent 19091S-433E column (30 m x 0.25 mm, 0.25 µm) was used. The initial temperature of the oven was 70°C, and the heating ramp was 15°C per minute until the maximum temperature of 290°C was reached, which was maintained for 6 minutes. The mobile phase was helium. The injector temperature was 250°C in split injection mode; the stream flow was 35cm/sec. The detector range of the mass spectrometer was 35-600 m/z, and 1 µl of sample was injected. The compounds were identified through a comparison with the NIST version 8.0 library database (National Institute of Standards and Technology, Gaithersburg, MD, USA).

HPLC-DAD

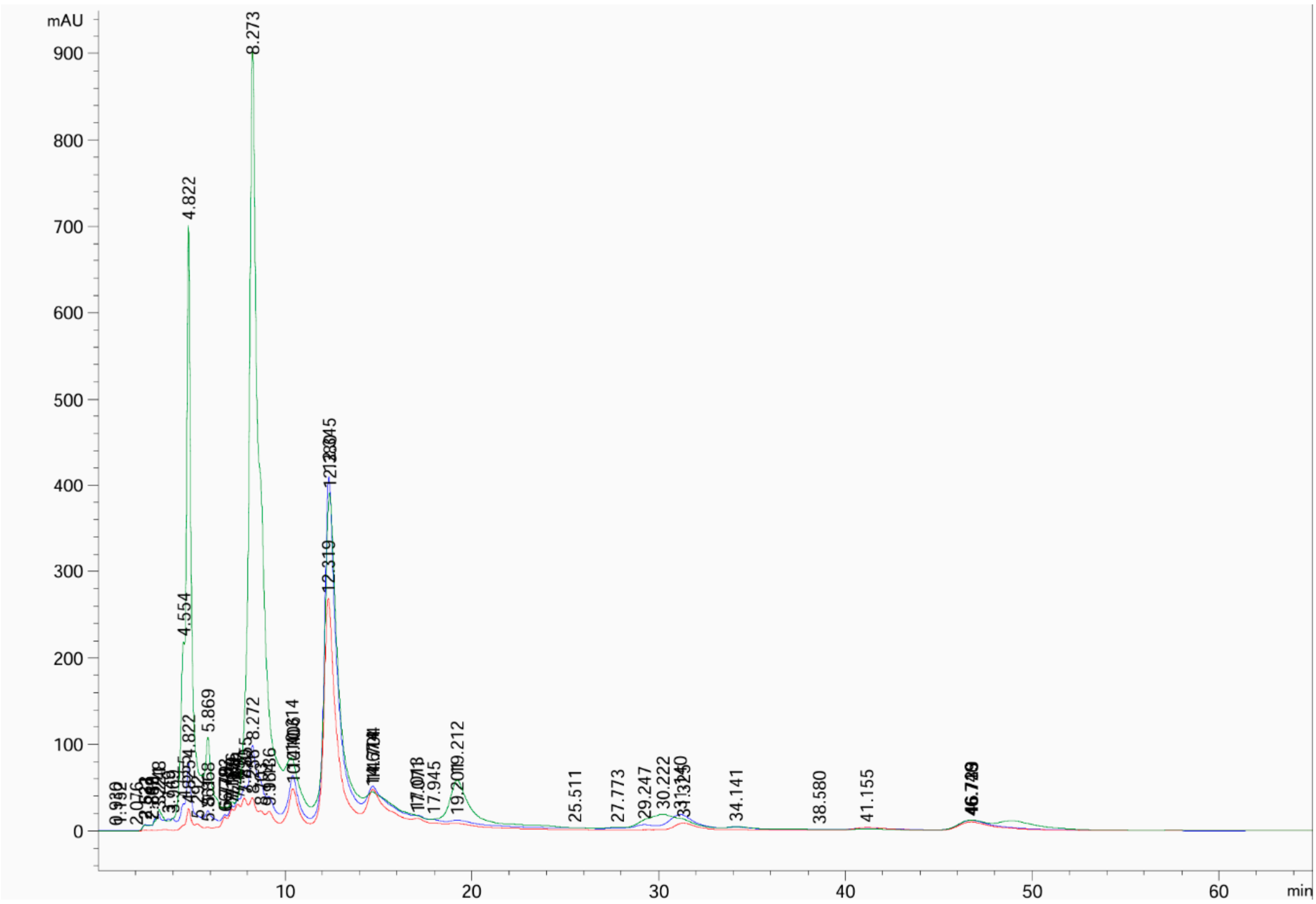

Chromatogram of the HPLC-DAD analysis of the chloroformic extract (CE) of propolis from Michoacán. Blue color: 254 nanometers, Green color: 280 nanometers, Red color: 365 nanometers. The chloroformic extract was injected at a concentration of 3 mg/mL with methanol-grade HPLC and a flow of 1 mL/min in the HPLC system model 1100 (Hewlett-Packard, Wilmington, USA) with the diode array detector (DAD) 1100 ChemStation A0903. A Discovery C-18 (250x4,6 mm) column was used with a pressure of 269 bar and a temperature range of 22°C – 23°C. The separation was isocratic, using a mobile phase of water–acetonitrile–methanol (50:25:25) and phosphoric acid (0.1%), and the detector was used at a wavelength of 260 nm with a full scan of 200–400 nm. The components of each extract were identified according to a comparison of the retention time of each peak and the UV spectrum with those of the standards. The following HPLC database standards were used: acacetin, baicalein, caffeine, catechin, catechol, chrysin, genistein, kaempferol, luteolin, myricetin, naringenin, naringin, genistein, pinocembrin, and quercetin. All standards were purchased from Sigma-Aldrich (USA).

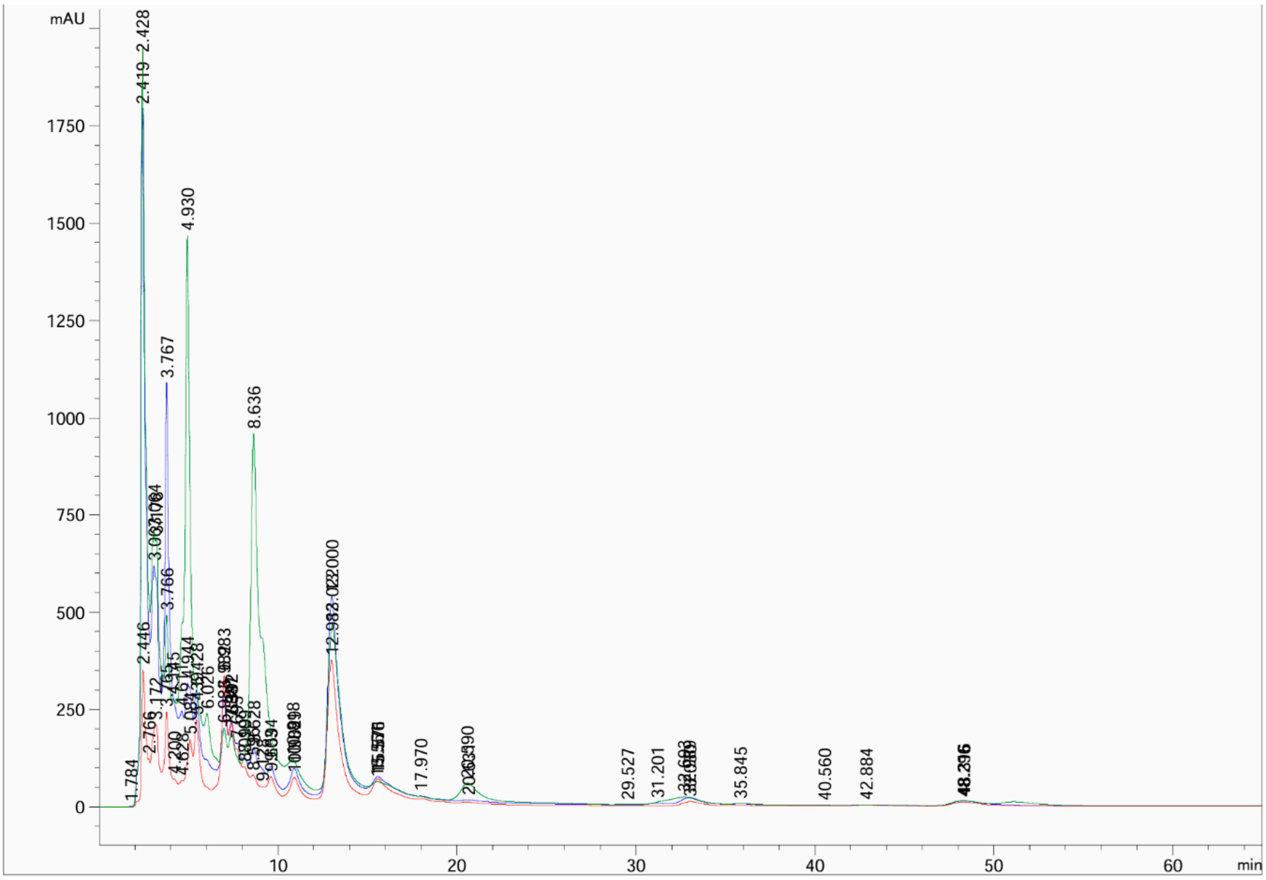

Chromatogram of the HPLC-DAD analysis of the methanolic extract (ME) of propolis from Michoacán. Blue color: 254 nanometers, Green color: 280 nanometers, Red color: 365 nanometers. The methanolic extract was injected at a concentration of 3 mg/mL with methanol-grade HPLC and a flow of 1 mL/min in the HPLC system model 1100 (Hewlett-Packard, Wilmington, USA) with the diode array detector (DAD) 1100 ChemStation A0903. A Discovery C-18 (250x4,6 mm) column was used with a pressure of 269 bar and a temperature range of 22°C – 23°C. The separation was isocratic, using a mobile phase of water–acetonitrile–methanol (50:25:25) and phosphoric acid (0.1%), and the detector was used at a wavelength of 260 nm with a full scan of 200–400 nm. The components of each extract were identified according to a comparison of the retention time of each peak and the UV spectrum with those of the standards. The following HPLC database standards were used: acacetin, baicalein, caffeine, catechin, catechol, chrysin, genistein, kaempferol, luteolin, myricetin, naringenin, naringin, genistein, pinocembrin, and quercetin. All standards were purchased from Sigma-Aldrich (USA).

HPLC-TOF-MS

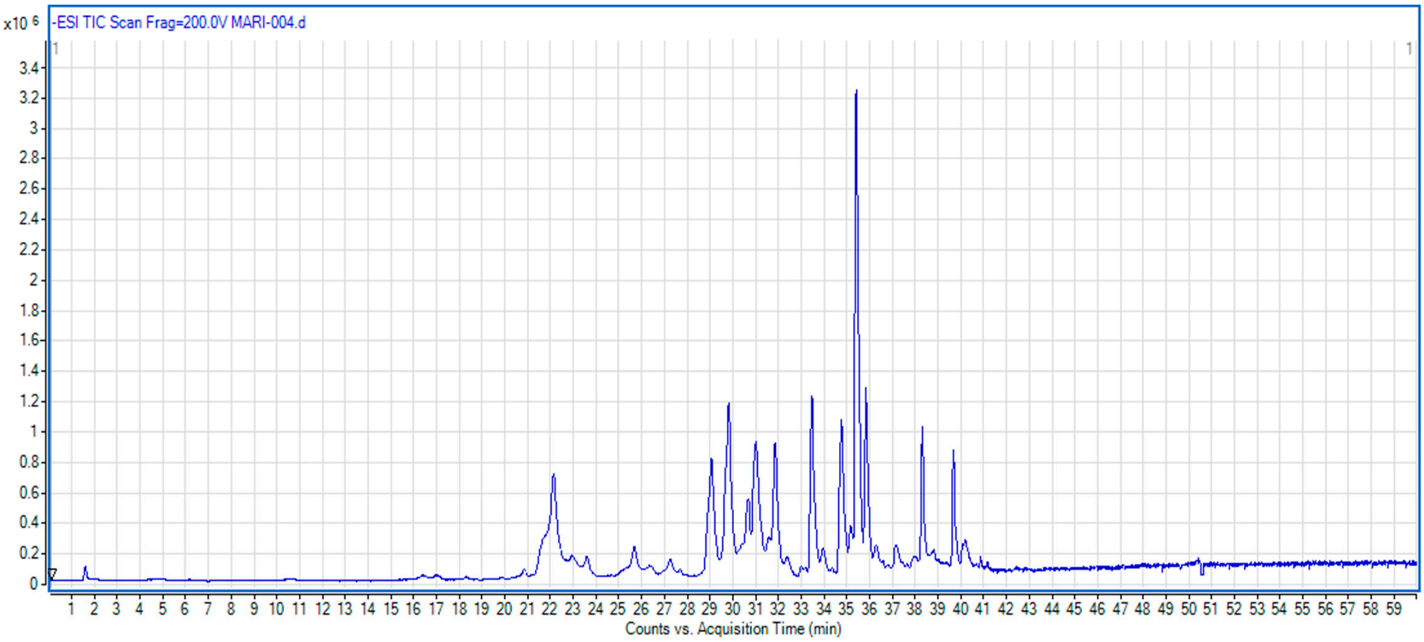

Chromatogram by HPLC-MS of the methanolic extract of propolis from Michoacan was performed using Agilent 1200 Infinity equipment, coupled to an Agilent 6230 TOF mass spectrometer with an Agilent Dual ESI source (ESISG14289023) and Mass Hunter Workstation software, Version B.05.01, Build 5.01.5125, operating in negative ionization mode. The capillary voltage was 4000 V; the dry gas temperature was 250 °C, with nitrogen used as the dry gas at a flow rate of 6 L/min; the nebulizer pressure was 60 psi, and the fragment was 200 V; the MS range was 50-1300 m/z; and the MS acquisition rate was 1 spectra/s. A Kinetex 2.6μ, C1800Å (150 x 2.1 mm) column (Phenomenex) maintained at 25°C and a two-line gradient mobile phase (solvents A and B) were used, where A = HPLC-grade water–HPLC-grade acetonitrile–formic acid (89:10:1) and B = HPLC-grade methanol–acetonitrile–formic acid (89:10:1). The first 3 min consisted of isocratic elution composed of 100% solvent A, followed by 3-11 min: 65% A, 35% B; 11-20 min: 55% A, 45% B; 20-35 min: 100% B. The flow rate was 0.2 mL/min, and the injection volume was 20 μL (3 mg/mL).

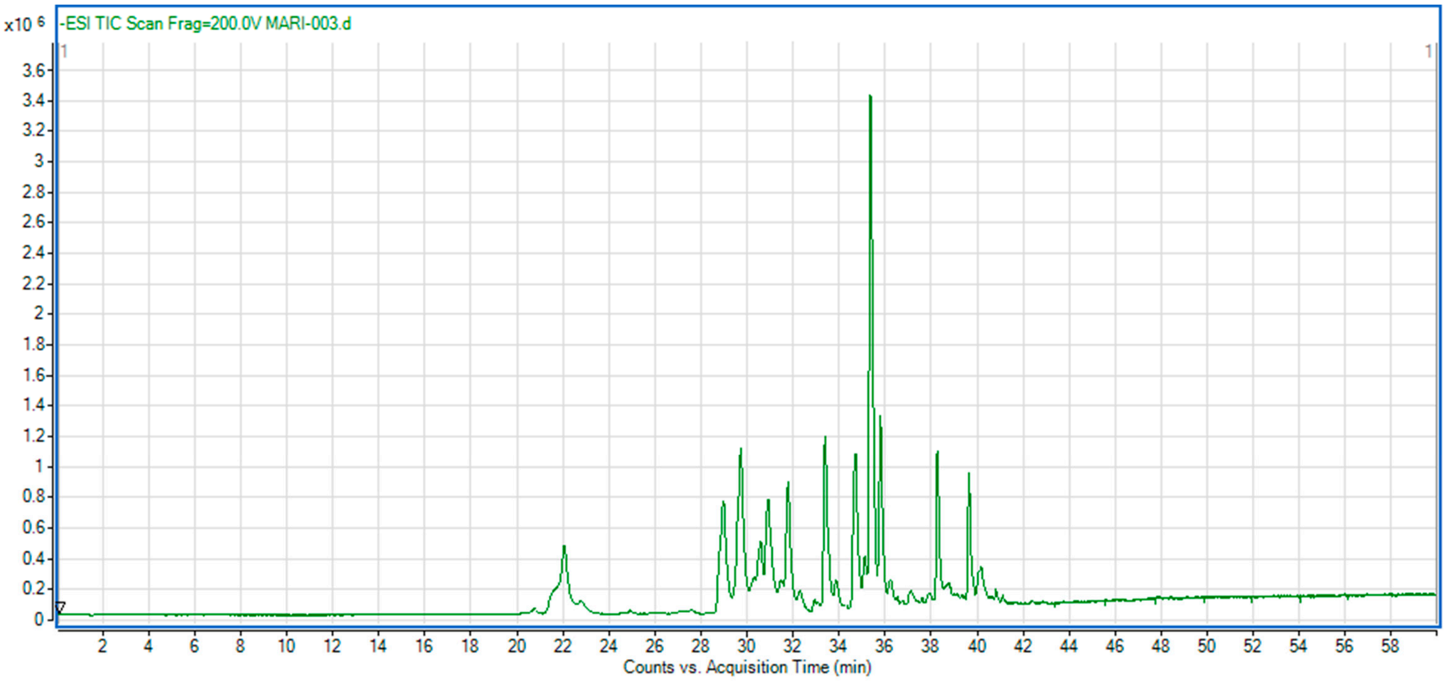

Chromatogram by HPLC-MS of the chloroformic extract of propolis from Michoacan was performed using Agilent 1200 Infinity equipment, coupled to an Agilent 6230 TOF mass spectrometer with an Agilent Dual ESI source (ESISG14289023) and Mass Hunter Workstation software, Version B.05.01, Build 5.01.5125, operating in negative ionization mode. The capillary voltage was 4000 V; the dry gas temperature was 250 °C, with nitrogen used as the dry gas at a flow rate of 6 L/min; the nebulizer pressure was 60 psi, and the fragment was 200 V; the MS range was 50-1300 m/z; and the MS acquisition rate was 1 spectra/s. A Kinetex 2.6µ, C1800Å (150 x 2.1 mm) column (Phenomenex) maintained at 25°C and a two-line gradient mobile phase (solvents A and B) were used, where A = HPLC-grade water–HPLC-grade acetonitrile–formic acid (89:10:1) and B = HPLC-grade methanol–acetonitrile–formic acid (89:10:1). The first 3 min consisted of isocratic elution composed of 100% solvent A, followed by 3-11 min: 65% A, 35% B; 11-20 min: 55% A, 45% B; 20-35 min: 100% B. The flow rate was 0.2 mL/min, and the injection volume was 20 µL (3 mg/mL).

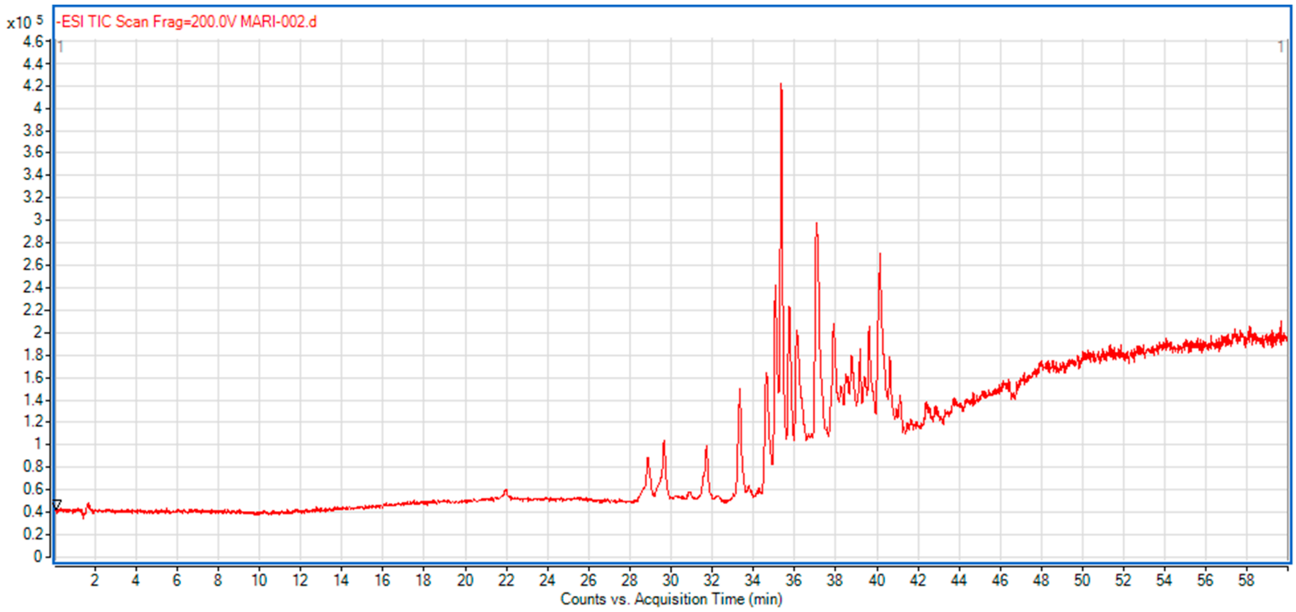

Chromatogram by HPLC-MS of the hexanic extract of propolis from Michoacan was performed using Agilent 1200 Infinity equipment, coupled to an Agilent 6230 TOF mass spectrometer with an Agilent Dual ESI source (ESISG14289023) and Mass Hunter Workstation software, Version B.05.01, Build 5.01.5125, operating in negative ionization mode. The capillary voltage was 4000 V; the dry gas temperature was 250 °C, with nitrogen used as the dry gas at a flow rate of 6 L/min; the nebulizer pressure was 60 psi, and the fragment was 200 V; the MS range was 50-1300 m/z; and the MS acquisition rate was 1 spectra/s. A Kinetex 2.6µ, C1800Å (150 x 2.1 mm) column (Phenomenex) maintained at 25°C and a two-line gradient mobile phase (solvents A and B) were used, where A = HPLC-grade water–HPLC-grade acetonitrile–formic acid (89:10:1) and B = HPLC-grade methanol–acetonitrile–formic acid (89:10:1). The first 3 min consisted of isocratic elution composed of 100% solvent A, followed by 3-11 min: 65% A, 35% B; 11-20 min: 55% A, 45% B; 20-35 min: 100% B. The flow rate was 0.2 mL/min, and the injection volume was 20 µL (3 mg/mL).
